# Supplementary material for: Mechanisms governing the pioneering and redistribution capabilities of the non-classical pioneer PU.1
Source: Nat Commun. 2020 Jan 21;11:402. doi: 10.1038/s41467-019-13960-2 (PMC6972792; doi:10.1038/s41467-019-13960-2)
Supplement: Supplementary file 7 — Source data [file 41467_2019_13960_MOESM7_ESM.zip › Source_Data/FigureS2/FigureS2B_MotifScanOutput/KmeansCluster7/homerResults/motif2.similar.html]

motif2

## Information for motif2

C
T
G
A
C
T
A
G
A
C
T
G
A
C
T
G
C
G
T
A
C
G
T
A
T
C
A
G
A
G
T
C
  
Reverse Opposite:  

C
T
A
G
A
G
T
C
A
C
G
T
A
C
G
T
A
G
T
C
A
G
T
C
A
G
T
C
A
G
C
T
  

|  |  |
| --- | --- |
| p-value: | 1e-499 |
| log p-value: | -1.151e+03 |
| Information Content per bp: | 1.874 |
| Number of Target Sequences with motif | 2047.0 |
| Percentage of Target Sequences with motif | 63.77% |
| Number of Background Sequences with motif | 9817.9 |
| Percentage of Background Sequences with motif | 22.46% |
| Average Position of motif in Targets | 102.9 +/- 54.1bp |
| Average Position of motif in Background | 99.2 +/- 74.9bp |
| Strand Bias (log2 ratio + to - strand density) | -0.3 |
| Multiplicity (# of sites on avg that occur together) | 1.07 |
| Motif File: | file (matrix) reverse opposite |

### Similar de novo motifs found

|  |  |  |  |  |  |  |  |
| --- | --- | --- | --- | --- | --- | --- | --- |
| Rank | Match Score | Redundant Motif | P-value | log P-value | % of Targets | % of Background | Motif file |
| 1 | 0.771 | G C T A C G A T C G T A C T A G C T A G T C A G T G C A C G T A T A C G A C G T T A G C C T G A C T A G | 1e-118 | -273.304151 | 14.77% | 3.90% | motif file (matrix) |
| 2 | 0.935 | C G T A A T C G A T C G A C T G C G T A C G T A A C T G | 1e-66 | -152.494106 | 25.45% | 13.50% | motif file (matrix) |
| 3 | 0.835 | C T A G T A C G A C T G A C T G G T C A C G T A T C A G A G T C C A G T | 1e-53 | -122.602327 | 7.35% | 2.08% | motif file (matrix) |
| 4 | 0.789 | G A C T C T A G T A G C A G C T G A C T A G T C A T G C T A G C C G A T A T G C | 1e-13 | -31.276683 | 8.01% | 4.77% | motif file (matrix) |
